# A scoping review of the measurement and analysis of frailty in randomised controlled trials

**DOI:** 10.1093/ageing/afae258

**Published:** 2024-11-21

**Authors:** Yanhe Sun, Miles D Witham, Andy Clegg, Rod S Taylor, Grace Dibben, David McAllister, Peter Hanlon

**Affiliations:** School of Health and Wellbeing, University of Glasgow, Glasgow, UK; AGE Research Group, Translational and Clinical Research Institute, Faculty of Medical Sciences, Newcastle University, Newcastle upon Tyne, UK; NIHR Newcastle Biomedical Research Centre, Newcastle upon Tyne NHS Foundation Trust, Cumbria Northumberland Tyne and Wear NHS Foundation Trust and Newcastle University, Newcastle upon Tyne, UK; Academic Unit for Ageing & Stroke Research, University of Leeds, Bradford Teaching Hospitals NHS Foundation Trust, Bradford, UK; School of Health and Wellbeing, University of Glasgow, Glasgow, UK; MRC/CSO Social and Public Health Sciences Unit, University of Glasgow, Glasgow, UK; Robertson Centre for Biostatistics, University of Glasgow, Glasgow, UK; School of Health and Wellbeing, University of Glasgow, Glasgow, UK; School of Health and Wellbeing, University of Glasgow, Glasgow, UK; School of Health and Wellbeing, University of Glasgow, Glasgow, UK

**Keywords:** frailty, trials, outcomes, randomised controlled trials, interventions, systematic review, older people

## Abstract

**Background:**

Frailty is of increasing interest in trials, either as a target of intervention, as an outcome or as a potential treatment modifier. However, frailty measurement is often highly variable. This scoping review assessed how frailty is quantified in randomised controlled trials (RCTs), in what context and for what purposes.

**Methods:**

We searched five electronic databases for RCTs in which frailty was measured among trial participants. We extracted data on intervention type, the frailty measure used and the purpose for which frailty was assessed. We then compared these data according to reasons for frailty assessment.

**Results:**

We identified 415 RCTs assessing frailty across a range of interventions. Frailty was used to define the target population (166 trials), as an outcome (156 trials), as an effect modifier examining interaction of frailty on treatment effect (61 trials), as a purely descriptive characteristic (42 trials) or as a prognostic marker examining the impact of frailty on future health outcome (78 trials). The trials used 28 different measures of frailty (plus 29 additional trial-specific measures). The frailty phenotype model was the most common overall (164 trials), for defining the target population (90/166 trials) and as an outcome (81/156 trials). The cumulative deficit model frailty index was also frequently used (102 trials) and was most common among trials assessing treatment effect modification (21/61 trials).

**Conclusion:**

Frailty measurement in RCTs is highly variable. Understanding the properties of respective frailty measures and how these relate to frailty as encountered in clinical practice is a priority to ensure that trial findings can inform healthcare delivery for people living with frailty.

## Key Points

- Frailty measurement in trials is highly variable, with >30 different measures used.
- Measure used to identify frailty tends to vary depending on the reason frailty is being assessed and the context of the trial.
- Consistency in measurement, defining transferability of measures and access to individual-level data may aid evidence synthesis.

## Introduction

Frailty is an age-related condition of increased vulnerability to decompensation in response to physiological stress [1]. Research on frailty has grown exponentially over the past two decades. Following the initial descriptions of the frailty phenotype model and the cumulative deficit model of frailty (both published in 2001) [2, 3], a wide range of tools and measures have been developed, based on diverse approaches and theoretical underpinnings [4]. As understanding of frailty as a dynamic and potentially modifiable state has developed, so too has interest in interventions to delay or reverse frailty [5]. Interest in frailty has also expanded widely beyond its origins in geriatric medicine, with exploration of the implications of frailty for treatment decisions in diverse clinical areas (including medical and surgical specialties, allied health disciplines and community health services) [6, 7]. This has led to frailty being assessed in a range of randomised controlled trials, either as a target for intervention, to define subgroups of participants or as an outcome in itself. However, the approach to assessing and reporting frailty in trials varies widely.

There remains no ‘gold standard’ or universally recommended measure to assess frailty, neither in research settings nor in clinical practice. Various measures have demonstrated validity in identifying groups of people at increased risk of important outcomes [8–10]. However, it is well established that the individuals identified as living with frailty will vary considerably depending on the measure used [11, 12]. In the context of randomised controlled trials, differences in the assessment and reporting of frailty may have particular significance, as trials ultimately seek to inform individual treatment decisions. Inferences made from trials using one frailty measure may not be applicable to people living with frailty identified using a different measure.

This scoping review systematically identifies randomised controlled trials (RCTs) that have measured frailty. We aim to describe (1) what measures have been used to assess frailty in these trials, (2) for what purposes frailty has been assessed within these trials and (3) whether the choice of frailty measure varies depending on the condition/target population of the trial or the purpose of frailty assessment.

## Methods

We conducted this scoping review according to a pre-specified protocol (https://doi.org/10.5281/zenodo.7925090) and report our findings according to the Preferred Reporting Items in Systematic Reviews and Meta-analyses—Scoping Reviews Extension (PRISMA-ScR) [13].

### Eligibility criteria

The exposure of interest was frailty in adults (>18 years old). Studies were eligible for inclusion regardless of the measure used to assess frailty as long as the study specified that the measure used was intended to capture frailty among trial participants. Studies had to describe or specify the tool used within the manuscript or the study protocol. We excluded studies that defined frailty exclusively based on setting (e.g. nursing home residents or people admitted to hospital departments specialising in older people).

We included RCTs (of any design). Observational, quasi-experimental studies and intervention studies without randomisation were excluded. Secondary analyses of RCT data were included where these analyses assessed frailty within the trial participants. To maximise comprehensiveness, we included trials irrespective of their intervention, comparator and outcomes.

We excluded published abstracts, trial protocols and grey literature. Systematic reviews were not eligible for inclusion.

### Information sources

We searched five electronic databases [Medline, Embase, Cumulated Index to Nursing and Allied Health Literature (CINAHL), Cochrane Central Register of Controlled Trials (CENTRAL) and Web of Science Core Collection] using terms for ‘frailty’ and ‘randomised controlled trials’. We used the Cochrane collaboration validated search terms for randomised controlled trials (maximising sensitivity). Full search terms are shown in the Supplementary Appendix. Our search was conducted to March 2023 and was limited to articles published after 2001 (the year of publication of the both the frailty phenotype and frailty index models for identifying frailty).

Two independent reviewers (Y.S. and P.H.) screened all identified titles and abstracts. Full texts of all potentially relevant records were screened for eligibility. Disagreements between reviewers were resolved by consensus involving a third reviewer where required.

We supplemented database searches by hand-searching reference lists of relevant systematic reviews as well as performing forward citation searching (using Web of Science) of relevant articles.

### Data extraction

Two independent reviewers (P.H. and Y.S.) extracted data from each of the included trials. We extracted bibliographic details (author, year, reference), trial details (condition of interest or target population, type of intervention, number of participants, mean age, sex distribution), details of frailty assessment (measure of frailty used in the trial, stated purpose of frailty assessment; e.g. identifying target population, defining subgroups or as an outcome) and whether the trial performed subgroup analyses by frailty status. We did not assess risk of bias of the included studies [14].

### Data synthesis

We synthesised the available data by producing a descriptive summary of the results. First, we grouped trials by disease area or target population. Within each group, we assessed the relative frequency of each frailty measure, as well as the purpose for which frailty was assessed. The latter was split into five categories—identifying target population, trial outcome, assessing treatment effect modification, analysing properties of frailty (other than effect modification) and describing frailty as a baseline characteristics without further analysis (these last three categories are by design mutually exclusive). We then cross-tabulated each frailty measure with the purpose for frailty assessment, both overall and within specific disease areas/target populations.

## Results

### Included trials

**Figure 1:**
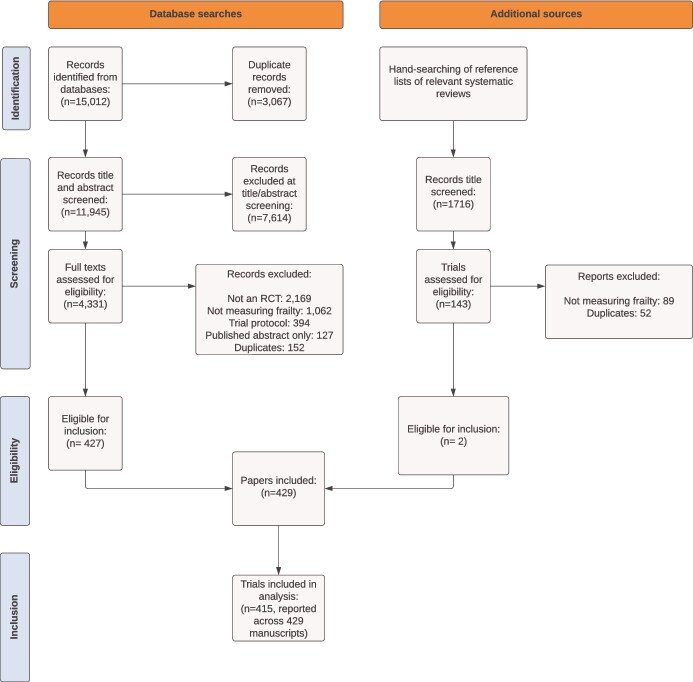
PRISMA Diagram of Study Identification and Inclusion.

We identified 429 eligible manuscripts describing 415 unique RCTs (Figure 1). Characteristics of the included trials are summarised in Table 1.

**Table 1:** Characteristics of included trials

Characteristic	Number of trials
**Target population**	
Older people * People living with frailty* * Community-dwelling older people* * People living with pre-frailty* * Nursing home residents* * People with impaired mobility* * Hospitalised older people* * Multiple long-term conditions* * Sarcopenia* Cardiometabolic disease Cancer Cognitive/neurological disorders Musculoskeletal disorders Malnutrition Respiratory disorders Surgical Liver disorders Menopause/hormonal therapies Renal disorders Infection Depression	213 * 82* * 73* * 28* * 13* * 7* * 4* * 3* * 3* 81 43 29 15 8 8 5 4 3 3 2 1
**Intervention**	
Exercise Pharmacological Multicomponent (inc. exercise, nutrition and others) Multidisciplinary/Comprehensive Geriatric Assessment Nutritional Vaccination Surgical Chronic disease treatment strategies (e.g. blood pressure targets) Medication optimisation Complementary/alternative medicine Cognitive training	126 101 88 38 24 11 10 9 5 5 2
**Purpose of frailty assessment**	
Identifying target population Trial outcome Assessing treatment effect modification Describing baseline characteristic (only) Analysing properties of frailty (only)	166 156 61 42 78
**Frailty measure**	
Fried frailty phenotype Cumulative deficit frailty index Study-specific measure (unnamed but described within trial) Clinical frailty scale FRAIL scale Short physical performance battery^*^ Groningen frailty indicator Edmonton frailty indicator Study for osteoporotic fractures frailty measure International Myeloma Working Group frailty measure Physical performance test Tilburg frailty indicator Comprehensive geriatric assessment Geriatric-8 Vulnerable Elders Study-13 Kihon checklist Liver frailty index Based on indicator conditions Brief frailty index Evaluative frailty index for physical activity FRESH screening tool Strawbridge questionnaire Berg balance scale^*^ CESAM questionnaire Fondazione Italiana Lonfomi tool Identification of Seniors at Risk tool Modified 11-item frailty index Modified 5-item frailty index PRISMA-7	164 102 28 26 15 15 12 11 8 7 7 6 5 5 5 4 4 3 2 2 2 2 1 1 1 1 1 1 1

^*^These measures were not developed as frailty measures (rather as measures of physical function), but were used in the included studies to identify frailty. Abbreviations: CESAM = Centre of Excellence on Longevity Self-AdMinistered.

The number of RCTs reporting frailty had increased in recent years: 243/429 papers (56%) were published after January 2020; 130/429 (30%) were published between January 2015 and December 2019. These covered a diverse range of clinical areas and types of intervention (Table 1).

Most trials (213/415, 51%, including all of those published prior to 2015) evaluated interventions targeting ‘older people’ either defined purely by age or people with clinical syndromes associated with ageing such as sarcopenia, frailty, polypharmacy or increased risk of falls. Interventions included multidisciplinary interventions based on comprehensive geriatric assessment, exercise interventions, nutritional support or multicomponent interventions combining exercise, nutrition and a range of other components. More recently, trials focussed on specific disease areas have also measured frailty. These include trials in people with cardiovascular disease/diabetes (*n* = 81 trials), cancer (*n* = 43 trials), neurological/cognitive disorders (*n* = 29 trials), musculoskeletal (*n* = 15 trials) and trials of surgical interventions (*n* = 5 trials).

A total of 116 trials (28%) used a measure of frailty to define the trial target population, 156 (38%) trials considered frailty as an outcome (55 of which also used frailty to define the target population), 61 trials (15%) explored whether the efficacy of the intervention under investigation was modified by frailty, while the remaining trials used measures of frailty when describing the characteristics of the study population (but did not use frailty to define inclusion, 42 studies) or used trial data to assess properties of the frailty measure itself (such as prognostic significance or association with biomarkers, 78 studies).

### Frailty measures

A total of 29 different frailty measures were used across the 415 trials, plus an additional 28 measures which were ‘trial specific’ (i.e. frailty was not based on a specific measure, but identified based on explicit criteria described within the trial manuscript). The most frequently used measure was the frailty phenotype (164/415 trials, 37%) followed by the frailty index (102/415 trials, 23%), the clinical frailty scale (26/415 trials, 6%), the FRAIL scale (15/415 trials, 3%) and the short physical performance battery (SPPB) (15/415 trials, 5%). In addition, 29/415 trials (7%) used more than one frailty measure.

Trials for some disease areas used frailty measures that had been designed specifically for people with the index condition in question. For example, 7 out of 14 trials for people with multiple myeloma assessed frailty using the International Myeloma Working Group frailty measure. Each of the trials assessing frailty in people with cirrhosis used the liver frailty index (4/4 trials).

Some frailty measures were also specific to types of intervention. For example, 15 studies defined frailty using the SPPB, a measure of lower extremity function and mobility (therefore relying on an explicitly physical definition of frailty based on muscle strength and function). All these studies evaluated exercise interventions, either alone or as part of a multicomponent intervention. Seven other studies, which defined frailty using different measures, also measured the SPPB as a measure of physical function but did not use it to define frailty, drawing a distinction between frailty and physical function. The frailty phenotype and frailty index were used across many disease areas. Measures that explicitly covered multiple domains (e.g. physical, psychological, social ± cognitive, such as the Groningen frailty indicator or the Tilburg frailty indicator) were used across multiple different disease areas and intervention types, but most commonly in trials of multidisciplinary or multicomponent interventions (e.g. 10/12 trials using the Groningen frailty indicator). While there was wide variation in measurement, this suggests that more explicitly physical conceptualisations of frailty were used more commonly in trials targeting physical endpoints (e.g. through exercise interventions) while the use of broader, multidimensional measures was largely confined to multicomponent interventions.

### Frailty as the target population

**Figure 2:**
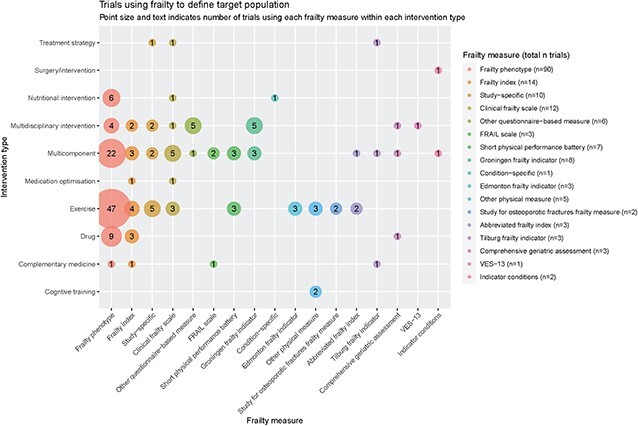
Trials using frailty to define target population.

We identified 166 trials using frailty (or pre-frailty) to identify the target population for the intervention. These are summarised in Figure 2. Exercise-based interventions were the most numerous, and 47/72 (65%) of exercise trials used the frailty phenotype. The frailty phenotype was also the most frequently used measure to identify the target population for nutritional interventions (6/8 trials) and multicomponent interventions incorporating exercise and nutritional support with or without additional components such as cognitive training (22/45 trials). In contrast, trials of multidisciplinary interventions (typically based on a comprehensive geriatric assessment model) that used frailty as inclusion criteria more frequently used the Groningen frailty indicator (5/21 trials) or other questionnaire-based measures (7/21 trials) that assessed frailty across multiple domains (including physical, social and cognitive).

### Frailty as an outcome

**Figure 3:**
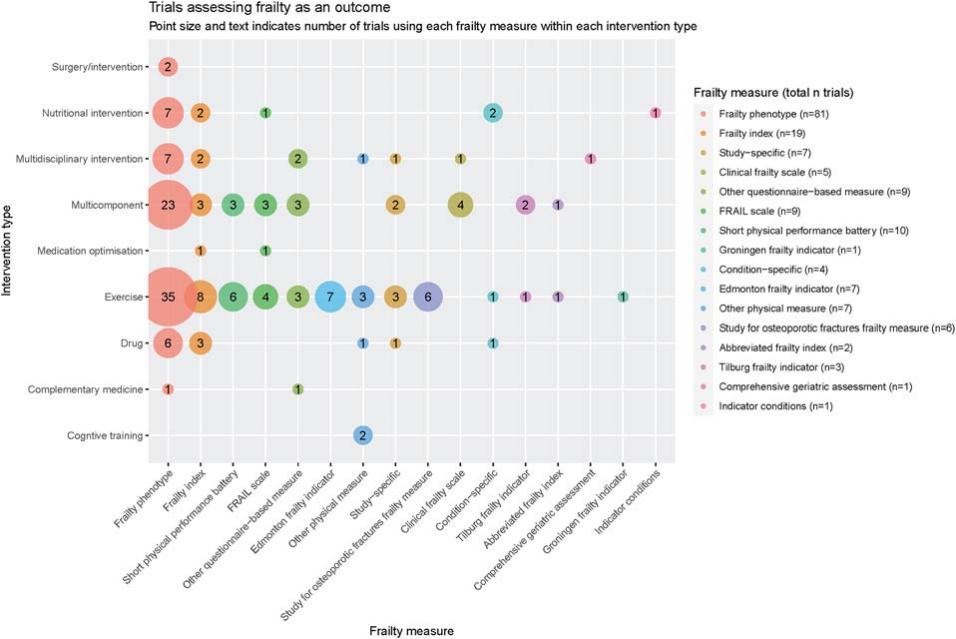
Trials assessing frailty as an outcome.

We identified 156 trials in which frailty was assessed as an outcome. These are summarised in Figure 3, by type of intervention. Frailty was specified as the primary outcome in 46/156 trials. Frailty phenotype was the most commonly used measure when assessing frailty as an outcome both overall (81/156 trials, 48%) and within categories of intervention.

### Frailty as an effect modifier

**Figure 4:**
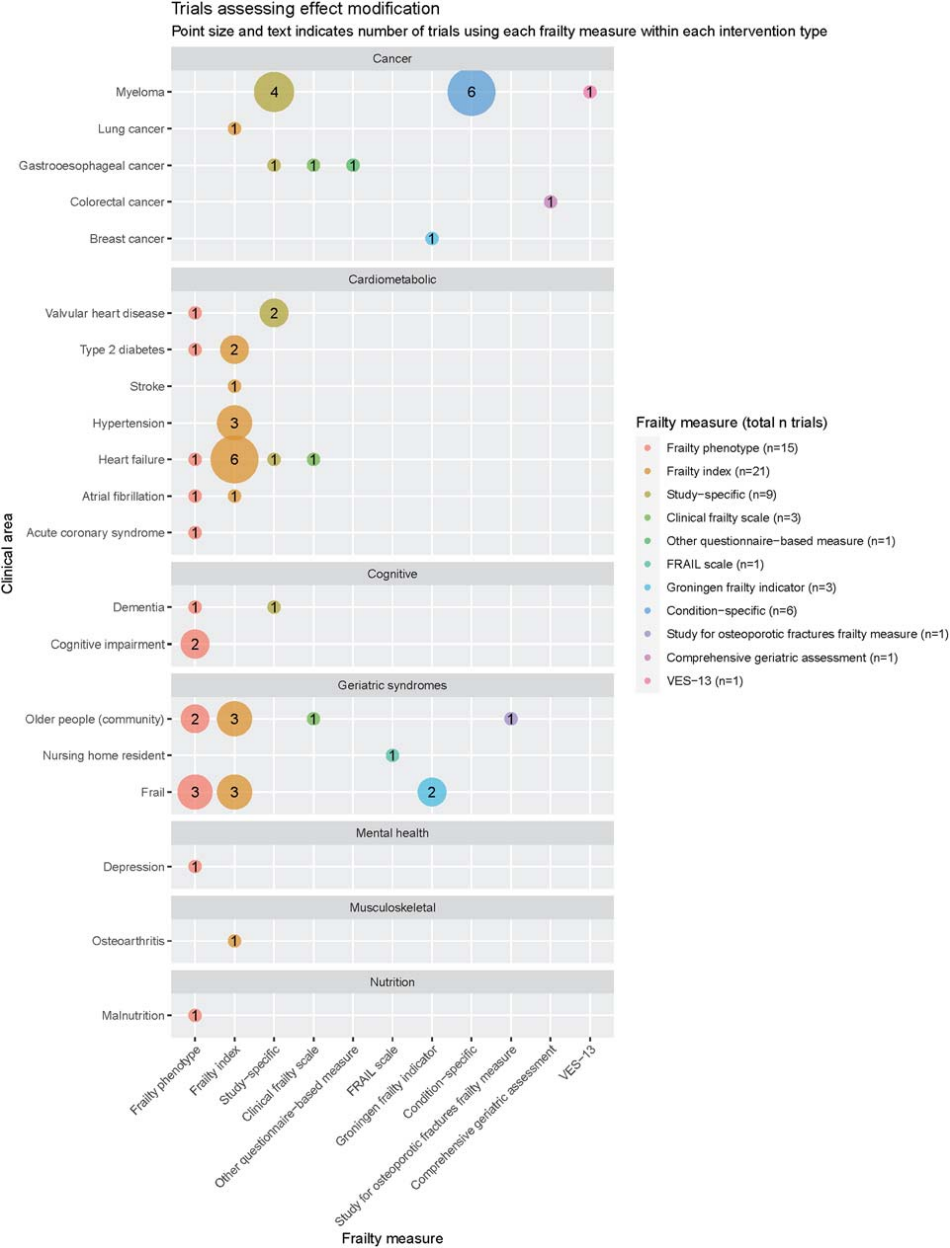
Trials assessing effect modification.

A total of 61 trials assessed whether the efficacy of the intervention varied by frailty status (Figure 4). The frailty index was the most commonly used measure (21/61 trials) followed by the frailty phenotype (15/61). Disease areas in which treatment effect modification by frailty had been assessed included drug trials for cardiovascular conditions including heart failure, hypertension and type 2 diabetes (most of which used a frailty index constructed *post hoc* from deficits collected at baseline); trials of chemotherapy regimens for myeloma (all of which either used the International Myeloma Working Group frailty measure or a study-specific measure incorporating age, comorbidity and some physiological parameters); as well as trials of multidisciplinary interventions focused on community-dwelling older people (frailty phenotype and frailty index in 5/16 and 6/16 trials, respectively).

## Discussion

This scoping review identified which frailty measures have been used within RCTs, in what contexts and for what purposes. Frailty quantification in trials was found to be highly variable, with >30 different measures having been used, although the frailty phenotype and frailty index were used in 37% and 23% of trials, respectively. The frailty phenotype was the most frequently used to define target populations for interventions and the most frequently used measure for frailty as an outcome, while the frailty index was the most used measure to assess whether frailty modified the efficacy of interventions. We found that frailty was assessed in trials for a diverse range of interventions including structured exercise, multidisciplinary clinical interventions, nutritional support and drug trials. While there was wide variation in frailty measurement in all areas, our findings suggest that frailty quantification varies by type of intervention (e.g. exercise interventions often using more exclusively physical definitions), clinical area (e.g. disease-specific frailty measures being used in trials for people with multiple myeloma or cirrhosis) and setting (e.g. multidomain measures explicitly incorporating social, psychological and cognitive function more commonly used in community-based multidisciplinary interventions).

The range of measures used in trials reflects the frailty literature more generally, in which a wide variety of measures are used in research and clinical practice and in which the frailty phenotype and frailty index predominate [4, 15]. These measures differ in their theoretical underpinnings and the range of measures and domains that are used to characterise frailty. Some draw a distinction between ‘physical frailty’ and other measures that variably include additional constructs such as cognition, psychological deficits or social vulnerability [4, 16]. Physical frailty is most frequently identified using the frailty phenotype, which is underpinned by a theoretical model of frailty in which dysregulation of interconnected physiological systems leads to disruption of homeostasis and vulnerability to decompensation [17]. Proponents of this model argue that interventions with multisystem effects (such as exercise) are likely to hold most promise in ameliorating physical frailty [17]. In developing guidance around the measurement of frailty, the European Medicines Agency specifically focussed on ‘physical frailty’ and proposed the SPPB as a potential proxy marker of frailty for this purpose [18]. However, physical models of frailty are often criticised for the exclusion of cognitive, psychological or social domains. Complex interventions targeting these aspects of frailty are therefore likely to be best evaluated using a model of frailty that includes these wider domains. Our findings that the frailty measures used to define target populations for different types of intervention largely reflect this division.

Different frailty measures identify different individuals as living with frailty [11, 19]. Furthermore, the way that frailty measures (such as the frailty phenotype) are operationalised and implemented can substantially influence the population identified [12]. While the choice of a given measure may be appropriate for a given context or intervention, the difference in population identified presents a challenge for meta-analysis and evidence synthesis, as considerable heterogeneity may result from studies using different definitions or specifying them differently [20]. To say an intervention ‘works’ or ‘does not work’ for people living with frailty is likely to depend on how frailty is defined. Efforts to map between measures of frailty offer potential solutions to these challenges [21]. Another is the use of individual participant data meta-analysis. Analysis of individual-level data can maximise consistency in the application of frailty definitions and allow harmonisation between definitions and the assessment of outcomes, thus allowing synthesis of frailty-specific inferences across studies. This is likely to be most feasible when studies assess frailty based on established models of frailty, rather than using study-specific measures or highly abbreviated measures as ‘proxies’ for frailty.

Our findings demonstrate the use of disease-specific frailty measures in areas such as multiple myeloma or cirrhosis. Such measures may have merit, as there may be specific stressors that are commonly encountered in a given clinical area, with corresponding markers that may optimally identify those at risk of decompensation [22]. However, these measures often classify frailty differently to ‘standard’ measures such as the frailty phenotype within the same individual (although, as mentioned above, this challenge is not limited to disease-specific measures) [23]. While some measures (such as the liver frailty index or the International Myeloma Working Group definition) are explicitly disease specific, some applications of ‘generic’ frailty measures in the included trials may be weighted towards the underlying condition of the trial. For example, the frailty index used in the SPRINT hypertension trial (based on the cumulative deficit model of frailty) has been criticised for relying too heavily on cardiovascular deficits and may not reflect the multisystem dysfunction that the frailty index is designed to reflect [24, 25]. A similar observation can be made of other applications of the cumulative deficit model to baseline trial data, many of which rely heavily on cardiovascular deficits or disease-specific clinical measures [26, 27]. These studies were, in general, secondary analyses seeking to explore whether frailty modified the efficacy of treatments. While the selection of multiple deficits related to the index condition likely reflects pragmatic decisions when applying a flexible approach such as the frailty index to trial data that were not collected for that purpose, it does limit applicability of claims regarding the representation of frailty in these trials or whether interventions are similarly effective across the frailty spectrum [28]. Frailty identified in this way may not necessarily reflect frailty as it is understood in clinical practice, where these treatments are used. In this context, research into the transferability of different frailty measures (e.g. through the development of mapping equations between definitions using a reference standard frailty measure as the anchor point) has potential to increase the applicability of frailty recommendations derived from trials.

There is growing recognition that frailty is a dynamic and potentially modifiable state [5, 29]. It is therefore important to understand what interventions may slow or reverse frailty or mitigate its impact. Most of the frailty measures that we identified as outcomes were not originally developed as outcome measures, but as tools to stratify populations or identify individuals at risk of adverse outcomes. There is a need to understand the psychometric properties of these measures, their potential responsiveness to intervention and what magnitude of change is clinically meaningful [30–32]. Conversely, some other measures (such as the short physical performance battery) were used in some studies to identify ‘frailty’ but were designed to measure constructs that are quite distinct from frailty (i.e. physical performance). While their responsiveness and properties as trial outcomes are more established, and the short physical performance battery was recommended by the European Medicines Agency as a marker of physical frailty, many trials using these measures do not invoke ‘frailty’ when demonstrating changes in physical function. Whatever measure is selected to identify frailty, these are ultimately proxy markers for physiological vulnerability to decompensation in response to stressors. How well these markers capture this vulnerability is also likely to vary. Where trials use established measures, their validity can be evaluated in the context of the broader frailty literature. However, where studies identify frailty using their own measures, or tools developed for other purposes, it will be more difficult to judge to what extent these interventions truly ‘improve frailty’. Whether improvement in any of these measures translates to a corresponding reduction in the risk of clinical outcomes that exemplify such decompensation (e.g. falls, delirium, hospital admission) is an important question for future research. However, we would argue that using existing frailty measures based on established models of frailty (rather than developing new measures or using tools developed for other purposes) is most likely to advance knowledge in this field.

Strengths of this scoping review include a comprehensive search strategy (using validated filters for trials designed to maximise sensitivity), using multiple databases, conducted without language restriction and supplemented by additional hand-searching of relevant articles. However, we excluded grey literature and unpublished trials, which may result in publication bias. We adopted broad inclusion criteria allowing assessment of frailty measurement across a diverse range of RCTs. However, this breadth and scale meant that it was not feasible to assess in detail how each of the frailty measures were implemented within each individual trials. We therefore did not assess (for example) the consistency of the application of the frailty phenotype across the included trials. Different operationalisations of these measure can yield different results (e.g. of prevalence of frailty) [12]. These would be potential areas for future examination. In keeping with scoping review methodology, we did not assess the quality of the included studies. Our findings are descriptive and exploratory and observations (such as differenced in frailty quantification between disease areas or reason for assessment) were not assessed statistically. Finally, we did not assess the extent of trials involving older people that did not include a measure of frailty, which is a major concern for translating evidence from trials involving heterogeneous groups of older people into clinical practice. For example, a 2024 network meta-analysis of community-based complex interventions for older people identified that only 16 out of a total 129 included trials used a validated measure to identify frailty [33].

In conclusion, frailty measurement within RCTs is increasing rapidly, with a diverse range of measures being applied across various clinical areas and types of intervention. Thus, frailty is both an opportunity and a challenge for triallists if we are to understand what interventions work for people living with frailty, how frailty might be modified and the applicability of disease-specific treatments for people living with frailty. The wide variation in the measurement of frailty presents considerable challenges to the translation of these findings to clinical practice. Given the range of contexts in which frailty is assessed, reasons for its measurement and properties of different measures, agreement on a single optimal method of frailty measurement is unlikely. However, various approaches could greatly enhance the broader application of trial evidence for people living with frailty. These include minimising variation in measurement where possible, developing mapping equations between different measures to support translation and implementation of findings, refining the use of existing tools rather than encouraging the proliferation of frailty measures, and to promoting the availability and use of individual participant data for meta-analysis. There is therefore a need to build consensus on the relative merits of approaches to quantifying frailty, developing standards for the application of specific measures such as the frailty phenotype or the cumulative deficit frailty index, and for infrastructure to support data sharing.

## Supplementary Material

aa-24-1160-File007_afae258
