# Supplementary material for: A scoping review of the measurement and analysis of frailty in randomised controlled trials
Source: Age Ageing. 2024 Nov 21;53(11):afae258. doi: 10.1093/ageing/afae258 (PMC11581818; doi:10.1093/ageing/afae258)
Supplement: aa-24-1160-File007_afae258 [file aa-24-1160-file007_afae258.docx]

**Supplementary appendix: A scoping review of the measurement and analysis of frailty in randomised controlled trials**

Search strategy (Medline database, adapted for other databases)

1 Randomized controlled trial/

2 Controlled clinical study/

3 random$.ti,ab.

4 randomization/

5 intermethod comparison/

6 placebo.ti,ab.

7 (compare or compared or comparison).ti.

8 ((evaluated or evaluate or evaluating or assessed or assess) and (compare or compared or comparing or comparison)).ab.

9 (open adj label).ti,ab.

10 ((double or single or doubly or singly) adj (blind or blinded or blindly)).ti,ab.

11 double blind procedure/

12 parallel group$1.ti,ab.

13 (crossover or cross over).ti,ab.

14 ((assign$ or match or matched or allocation) adj5 (alternate or group$1 or intervention$1 or patient$1 or subject$1 or participant$1)).ti,ab.

15 (assigned or allocated).ti,ab.

16 (controlled adj7 (study or design or trial)).ti,ab.

17 (volunteer or volunteers).ti,ab.

18 human experiment/

19 trial.ti.

20 or/1-19

21 Frailty/

22 frail*.tw.

23 21 or 22

24 20 and 23
